# Modifying soil bacterial communities in saline mudflats with organic acids and substrates

**DOI:** 10.3389/fmicb.2024.1392441

**Published:** 2024-04-19

**Authors:** Xiaoyu Liu, Liang Zhong, Ruixue Yang, Huiyan Wang, Xinbao Liu, Wei Xue, He Yang, Yixin Shen, Jianlong Li, Zhengguo Sun

**Affiliations:** ^1^College of Agro-grassland Science, Nanjing Agricultural University, Nanjing, China; ^2^School of Life Science, Nanjing University, Nanjing, China; ^3^Nanjing University (Suzhou) High and New Technology Research Institute, Suzhou, China

**Keywords:** mudflat soil, organic acid, biological substrate, bacterial community structure, soil microbial enhancement

## Abstract

**Aims:**

The high salinity of soil, nutrient scarcity, and poor aggregate structure limit the exploitation and utilization of coastal mudflat resources and the sustainable development of saline soil agriculture. In this paper, the effects of applying exogenous organic acids combined with biological substrate on the composition and diversity of soil bacterial community were studied in moderately saline mudflats in Jiangsu Province.

**Methods:**

A combination of three exogenous organic acids (humic acid, fulvic acid, and citric acid) and four biological substrates (cottonseed hull, cow manure, grass charcoal, and pine needle) was set up set up on a coastal saline mudflat planted with a salt-tolerant forage grass, sweet sorghum. A total of 120 kg ha^−1^ of organic acids and 5,000 kg ha^−1^ of substrates were used, plus two treatments, CK without application of organic acids and substrates and CK_0_ in bare ground, for a total of 14 treatments.

**Results:**

No significant difference was found in the alpha diversity of soil bacterial community among all treatments (*p* ≥ 0.05), with the fulvic acid composite pine needle (FPN) treatment showing the largest increase in each index. The beta diversity differed significantly (*p* < 0.05) among all treatments, and the difference between citric acid–grass charcoal (CGC) and CK treatments was greater than that of other treatments. All treatments were effective in increasing the number of bacterial ASVs and affecting the structural composition of the community. Citric acid–cow manure (CCM), FPN, and CGC treatments were found to be beneficial for increasing the relative abundance of *Proteobacteria*, *Chloroflexi*, and *Actinobacteria*, respectively. By contrast, all treatments triggered a decrease in the relative abundance of *Acidobacteria*.

**Conclusion:**

Among the 12 different combinations of exogenous organic acid composite biomass substrates applied to the coastal beach, the CGC treatment was more conducive to increasing the relative abundance of the salt-tolerant bacteria *Proteobacteria*, *Chloroflexi* and *Actinobacteria*, and improving the community structure of soil bacteria. The FPN treatment was more conducive to increase the species diversity of the soil bacterial community and adjust the species composition of the bacterial community.

## Introduction

As indispensable decomposers in the soil ecosystem, microorganisms not only participate in important material transformation and nutrient cycling processes in soil but also regulate nutrient uptake by aboveground plants (Gu et al., 2022). Soil microorganisms are strongly influenced by extreme soil environments when utilizing metabolic activities to alter soil physicochemical properties (Dong et al., 2022), and their community structure, spatial distribution, and biodiversity are regulated by the effects of various factors such as soil nutrients, hydrometeorological conditions, salinity, vegetation type, and land-use practices (Valéria et al., 2021). Poor physical structure, nutrient deprivation and high basal salinity of coastal beach soils inhibit soil microbial activity and affect microbial community diversity (Albdaiwi et al., 2019; Zhao et al., 2022). The main pressure on microbial survival in coastal mudflat soils is salinity, and microbial abundance and activity are negatively correlated with salinity (Siddikee et al., 2011; Wu et al., 2015; Singh, 2016). Related studies have shown that soil fungi are more resistant to salinity stress than soil bacteria due to their chitin cell walls and different energy generation systems from bacteria (Strickland and Rousk, 2010; Rath et al., 2016). In a study by Sun et al. (2021) on the abundance and vertical distribution of soil microbiomes in coastal saline soils under different amelioration measures, it was similarly found that soil bacteria had a more pronounced response to salt concentration, with significantly higher abundance than fungi and archaea. In addition, soil bacteria are involved in the ecological processes of material decomposition and nutrient cycling, and their metabolic activities play an important role in promoting the mineralization of organic matter and humus formation in mudflat soils, which is one of the main factors in restoring and maintaining the productivity of saline soils (Bardgett and van der Putten, 2014; Xiang et al., 2021), so bacterial communities are to a large extent determines the sustainable productivity of beach agroecosystems (Bender et al., 2016).

Plant root exudates in mudflat soils with organic acids as the main components induce and enrich the mass reproduction of some bacterial communities, thus changing the composition and diversity of rhizospheric bacterial communities and then triggering changes in the structure and function of bacterial communities in saline alkali soil (Pei et al., 2017; Zheng et al., 2018). Exogenously applied organic acid–based biological substrates can cause directional changes in the internal structure and composition of the community through their unique physicochemical properties and nutrient composition; they take advantage of bacterial preferences for different living environments and energy substances, thus promoting the ripening of mudflat soil (Mao et al., 2022). Related studies have shown that organic acids and their composite products can not only provide a large amount of nutrients for soil microorganisms, improve soil microbial community diversity, but also improve soil microenvironment through the formation of soil aggregates, providing independent habitats for different microbial groups (Compant et al., 2009). Humic acid compound fertilizer has a sustained effect on microbial community changes at different plant growth stages, and the promotional effect on the increase in the abundance of beneficial fungal and bacterial communities differs at various times (Liu et al., 2019). Humic acid-rich organic amendments have a significant promotional effect on the increase in the abundance of microorganisms involved in the process of carbon and nitrogen cycling, such as *aerobic_ammonia_oxidation*, *aerobic_chemoheterotrophy*, and *nitrification*, and they have an important effect on the functional diversity of microorganisms (Guo et al., 2022). Compared with humic acid, fulvic acid has a lower molecular weight, and higher oxygen and lower carbon make fulvic acid contain more acidic functional groups (especially carboxyl groups), which will be more favorable to reduce soil salinity and promote soil microbial uptake and utilization (Wang, 2008; Zhang and Katayama, 2012; Martinez et al., 2013). Citric acid can enhance the carbon source metabolism ability of soil microbial communities, strengthen carbon turnover efficiency in saline alkali soil (Su et al., 2022), and enhance microbial activity. These advantages affect the internal composition of bacteria and fungi and promote their secretion of extracellular enzymes, thereby facilitating the improvement of soluble nutrient conversion efficiency in saline soils (Macias-Benitez et al., 2020).

Changes in soil physicochemical properties triggered by biological substrate application, such as changes in soil organic carbon, soil pH, and electrical conductivity, indirectly drove adjustments in the structure of soil bacterial and fungal communities, enhanced the relative abundance of bacteria and fungi associated with available nitrogen and phosphorus transformations, and reduced the proportion of pathogenic genera in soil (Ding and Li, 2022; Wang Z. H. et al., 2022). These adjustments have a positive effect on soil structure improvement and microecological environment promotion (Wang Z. H. et al., 2020). Wang Z. J. et al. (2022) showed that the application of organic materials, such as cow manure, biochar, and straw, effectively increased the bacterial abundance and community diversity of coastal saline alkali soils, and they also showed different transformation directions in the structural composition of the bacterial community. Acid-modified biochar significantly increased the relative abundance of *Acidobacteria*; as well as the number of bacteria in specific families, such as Pseudomonadaceae and Sphingobacteriaceae, which have strong ecological connections with C, N, and P cycling and organic matter degradation, and had a strong effect on mitigating soil salinity stress and neutralizing soil pH (Soothar et al., 2021). Pine forest litter exhibited differences in fungal and bacterial biomass, structure, and functional diversity and significantly affected the content and activity of soil enzymes (Vuong et al., 2020; Picariello et al., 2021).

Although domestic and international research involved the influence of different types of biological substrates or organic acid addition on soil microorganisms in saline alkali areas, the utilization mode is mostly the addition of a single organic material. However, studies have found that natural biological substrates such as plant litter, livestock manure, and agricultural by-products have an important impact on improving soil structural stability and accelerating salt leaching (Farrell et al., 2011; Chen et al., 2019). The groove-like microstructure of pine needles has good water collection properties, which helps the uniform distribution and adhesion of exogenous organic acids on its surface, and the proportion of soluble nutrients in pine needles is large, which can rapidly increase the content of quick-acting nutrients in the soil, and its salinity reduction in saline soil remediation process has an obvious effect on the reduction of salts. The study of Kusvuran et al. (2021) showed that cow dung and humic acid application can reduce the salinity of soil by reducing the damaging effects of salt stress by decreasing the uptake of Cl^−^ and Na^+^ with enhancing the uptake of K^+^ and Ca^2+^, and that a decrease in malondialdehyde content had a favorable effect on alleviating the oxidative stress response of the crop.

The composite application of organic acids and bio-based materials, with their unique physical structure and chemical function, effectively slowed down the leaching process of organic acid components, overcame the effects of soil salinity and other adversities in a short period of time, increased the saline and alkaline nutrient reservoirs, accelerated the decomposition of the organic matter in the soil and the bio-based materials, and released the necessary nutrients for soil microbial uptake and utilization (Wang, 2021; Xiao et al., 2022). If scientifically applied to mudflat soils not only promotes the growth and metabolism of soil bacteria, but also positively influences bacterial community diversity (Wang et al., 2015; Matteo et al., 2022). However, at present, relatively few studies have been conducted on the regulation of bacterial community composition and diversity in saline alkali soils by organic material addition. Moreover, the effect of the combination of exogenous organic acids and biological substrates on the bacterial community structure in mudflat has not been reported. In this study, we chose the salt-tolerant forage sweet sorghum with strong resistance and good palatability as the test material (Hu et al., 2017), sampled the soil during the tasseling period of sweet sorghum, and analyzed the effects of different exogenous organic acid composite biobased treatments on the structural characteristics of soil bacterial communities through high-throughput sequencing technology, and derived the alpha diversity and beta diversity indices based on the sequencing results, and analyzed the effects on the soil bacterial communities diversity under different The effects of the treatments on the diversity of soil bacterial communities were analyzed, and the species composition and differences of soil bacteria in different treatments were comparatively analyzed through species taxonomic level annotation. The purpose of this study is to explore the advantageous combinations of different organic acids and biological substrates to enhance the abundance of soil salt-tolerant microorganisms, to improve the status quo of microbial barrenness in coastal saline soils, and to provide scientific basis for the microecological improvement of soils in coastal mudflat areas.

## 1 Materials and methods

### 1.1 Experimental site

The experiment was carried out in a coastal saline soil in the strip mud reclamation area of Snare Town, Dongtai City, Jiangsu Province, China (N 32°50′01′′, E 120°56′43′′). The soil type in this area is silty loam and contains 20.5% sand, 8.0% clay, and 71.5% silt (0–20 cm). The specific physicochemical properties are shown in Table 1. The area has a subtropical monsoon maritime climate with an average annual temperature of 16.3°C and an average annual precipitation of 1,024 mm, which mainly occurs in summer (Wang et al., 2023; Yang et al., 2023).

**Table 1:** Basic physical and chemical properties of primitive soil.

Items	Values	Items	Values
FC (%)	23.05 ± 0.16	OM (g kg^−1^)	8.77 ± 0.02
BD (g cm^−3^)	1.47 ± 0.04	TN (g kg^−1^)	0.45 ± 0.01
pH	9.16 ± 0.01	TP (g kg^−1^)	0.52 ± 0.02
EC_1:5_ (us cm^−1^)	492.00 ± 4.73	AN (mg kg^−1^)	22.18 ± 0.13
SS (g kg^−1^)	3.22 ± 0.04	AP (mg kg^−1^)	14.18 ± 0.02

FC, field water capacity; BD, soil bulk density; EC_1:5_ electrical conductivity of 1:5 soil:water extract; SS, soluble salt; OM, organic matter; TN, total nitrogen; TP, total phosphorus; AN, alkali-hydrolyzed nitrogen; AP, available phosphorus.

### 1.2 Experimental design

A field experiment was conducted from June 2021, to September 2021, with a total of 14 treatments set up: (1) HCM, supplemented with humic acid and cow manure; (2) HPN, supplemented with humic acid and pine needle; (3) HCH, supplemented with humic acid and cottonseed husk; (4) HGC, supplemented with humic acid and grass charcoal; (5) FCM, supplemented with fulvic acid and cow manure; (6) FPN, supplemented with fulvic acid and pine needle; (7) FCH, supplemented with fulvic acid and cottonseed hull; (8) FGC, supplemented with fulvic acid and grass charcoal; (9) CCM, supplemented with citric acid and cow manure; (10) CPN, supplemented with citric acid and pine needle; (11) CCH, supplemented with citric acid and cottonseed hull; (12) CGC, supplemented with citric acid and grass charcoal; (13) CK, without the addition of organic acids and biomasses; and (14) CK_0_, bare ground treatment. All treatments were replicated three times, and the application rates of organic acid and biomass were 120 and 5,000 kg ha^−1^, respectively, in all treatments. The sources and properties of organic acid materials are shown in Table 2. Cow manure was aerobically composted and fermented for about 40 days after wet and dry separation. Cottonseed husk was made from residual husk scraps of edible mushroom culture material. Grass charcoal was prepared from the accumulation of incompletely decomposed plant residues fermented in an overly wet and suspicious natural environment. Pine needles referred to those that had accumulated on the soil surface of lacebark pine forests and were collected after natural weathering and decomposition. The specific physical and chemical properties of these biomass materials are shown in Table 3.

**Table 2:** Sources and basic properties of organic acids.

Organic acid	Solubility	Structure	Appearance	pH	C (%)	H (%)	O (%)	N (%)
Humic acid	Slightly soluble in water	High molecular polymer	Black powder	6.82 ± 0.02	37.91 ± 0.24	3.46 ± 0.03	25.43 ± 0.24	0.61 ± 0.01
Fulvic acid	Soluble in water, acid, and alkali	High molecular polymer	Brown powder	5.26 ± 0.03	28.35 ± 0.18	4.23 ± 0.02	41.18 ± 0.17	3.24 ± 0.02
Citric acid	Highly soluble in water	Low molecular weight	White granule	1.47 ± 0.01	36.72 ± 0.10	4.77 ± 0.03	55.30 ± 0.19	0.18 ± 0.01

FC, field water capacity; BD, soil bulk density; EC_1:5_ electrical conductivity of soil:water extract at 1:5 ratio, SS, soluble salt; OM, organic matter; TN, total nitrogen; TP, total phosphorus; AN, alkali-hydrolyzed nitrogen; AP, available phosphorus. Humic acid and fulvic acid are provided by Xinjiang Shengda Party Biotechnology Co., Ltd (Xinjiang, China) and citric acid is produced by Weifang Ensign Industry Co (Shandong China).

**Table 3:** Sources and basic properties of biomass materials.

Biological substrates species	pH	Electrical conductivity (μs cm^−1^)	Organic matter (g kg^−1^)	Total nitrogen (g kg^−1^)	Total phosphorus (g kg^−1^)	Alkali-hydrolyzed nitrogen (mg kg^−1^)	Available phosphorus (mg kg^−1^)
Cottonseed hull	7.30 ± 0.03	1682.00 ± 15.52	297.91 ± 3.58	11.85 ± 0.63	2.95 ± 0.01	728.85 ± 8.24	776.70 ± 8.98
Pine needle	5.38 ± 0.02	1308.00 ± 6.66	407.85 ± 5.54	10.20 ± 0.09	0.66 ± 0.01	1374.46 ± 24.71	79.73 ± 1.87
Grass charcoal	5.08 ± 0.03	841.00 ± 9.07	244.12 ± 3.22	8.46 ± 0.02	0.79 ± 0.01	693.22 ± 16.13	45.82 ± 1.75
Cow manure	7.70 ± 0.03	1416.00 ± 8.14	345.79 ± 4.91	9.69 ± 0.04	2.36 ± 0.02	477.55 ± 6.69	519.76 ± 8.53

Cottonseed hull, pine needle, and grass charcoal were purchased from Shijiazhuang Nongyou Biotechnology Co. Ltd., and cow manure was provided by Jurong Lantian Bishui Biotechnology Co., Ltd. EC_1:5_, electrical conductivity of soil:water extract at 1:5 ratio; OM, organic matter; TN, total nitrogen; TP, total phosphorus; AN, alkali-hydrolyzed nitrogen; AP, available phosphorus. The three bio-based materials were purchased from Shijiazhuang Nongyou Biotechnology Co (Hebei, China).

The study was conducted in a plot test with a split-zone design and a plot arrangement of 10 m^2^ (4 m long and 2.5 m wide). The soil tillage layer (0–20 cm) was rototilled before the start of the experiment. In addition to the land preparation, drainage ditches (30 cm deep and 40 cm wide) were opened between the plots so that these ditches were connected to the drainage pipes at the edges of the fields to avoid seedling damage caused by waterlogging in the fields. A double layer of mulch was placed along a vertical depth of 0–30 cm close to the edge position around each plot to stop the migration of water salts, nutrients, and other substances between the plots. The base fertilizer application was 300 kg ha^−1^ of compound fertilizer (15% N, 15% P, and 15% K), which was applied during land preparation. The follow-up fertilizer was applied two times at 30 and 60 days after seedling emergence with 75 kg ha^−1^ of urea (46.4% N). The organic acid pellets (or powder) were mixed evenly with the biomass material and then spread into the plots and pulled back and forth with a rake to mix evenly into the topsoil. The sweet sorghum variety was Big Kahuna, which was planted in June 2021 via strip sowing with a row spacing of 40 cm and a sowing depth of 2 cm. The seedlings were set to a specification standard of 25 cm apart during the three-leaf period. During the growing season, timely prevention and elimination were carried out in conjunction with the occurrence of diseases, insects, and weeds in the field. Owing to the abundant rainfall during the growing season, no irrigation was carried out for moisture management, and the water in the field was removed in a timely manner.

### 1.3 Soil sampling methods

During the heading stage of sweet sorghum (90 days after emergence), soil samples were collected using the five-point method (“S” distribution) at a depth of 0–20 cm. The collected soil at five points was mixed evenly as a sample, with three replications for each treatment. After impurities were removed, the soil samples were placed in sterile sealed bags and quickly brought back to the laboratory in an ice box. The soil samples were mixed well and passed through a 2 mm-sterile sieve, and 5–10 g was taken and stored in sterile EP tubes in a −80°C refrigerator for soil microbial determination and analysis.

### 1.4 Soil DNA extraction, PCR amplification, and library construction

First, 0.5 g soil sample stored in a −80°C refrigerator was weighed, and nucleic acid was extracted using an OMEGA D5625-01 Soil DNA Kit (OmegaBio Tek, Norcross, GA, United States). The extracted soil DNA was detected by 0.8% agarose gel electrophoresis and quantified by a UV spectrophotometer. Then, the highly variable V3–V4 region of the bacterial 16S rRNA gene (Wang Z. et al., 2020), which was about 468 bp in length, was selected for sequencing conducted by Nanjing Personal Gene Technology Co., Ltd. (Nanjing, China). PCR amplification was performed using the following bacterial 16S rDNA V3–V4 region specific primers: 338\u00B0F (55′- ACTCCTACGGGGAGGCAGCA-3′) and 806 R (5′- GACTACHVGGGTWTCTAAT-3′). The PCR amplification program was as follows: pre-denaturation at 98°C for 30 s; 26 cycles of 98°C denaturation for 15 s, annealing at 50°C for 30 s, extension at 72°C for 30 s; and maintain at 72°C for 5 min. The PCR amplification products were detected by 2% agarose gel electrophoresis, and then the target fragments were recovered using an Axygen gel recovery kit (Soothar et al., 2021). The recovered products were subjected to fluorescence quantification with a Quant-iT PicoGreen dsDNA Assay Kit as the fluorescence reagent and a microplate reader (BioTek, FLx800, Agilent, United States) as the quantification instrument. Libraries were constructed using Illumina’s TruSeq Nano DNA LT Library Prep Kit (San Diego, CA, United States). On the basis of the fluorescence quantification results, each sample was mixed in the corresponding proportion in accordance with its sequencing quantity requirements, and machine sequencing and subsequent data processing were performed.

### 1.5 Data statistical analysis

The original sequence of the microbiome was processed using QIIME 2 (2019) software (Bolyen et al., 2019), and the merged ASV feature sequence was compared with the reference sequence in Silva database to obtain specific taxonomic information of ASV. Data analysis and image rendering were mainly carried out using QIIME 2 and R software packages (version 3.2.0). QIIME 2 software was used for the following purposes: to draw sparse curves to evaluate whether the current sample size could reflect the real situation of the changes in the structural characteristics of the bacterial community; to calculate the alpha diversity indices, such as Chao1, Shannon, and Faith’s PD, and the Good’s coverage of the soil bacterial community; and to present the soil bacterial community abundance, diversity, and coverage in the form of box plots. Beta diversity was analyzed on the basis of Bray–Curtis distance to characterize the developmental distances between soil bacterial communities by nonmetric multidimensional scaling (NMDS) and statistically tested using permutational multivariate analysis of variance (PERMANOVA). The number of unique versus shared ASVs between different treatments was counted using petal plots generated by Venn Diagram in R package. MetagenomeSeq analysis was used to statistically compare the abundance of taxonomic groups at the phylum and genus levels between treatments, and linear discriminant analysis (LDA effect size, LEfSe) was used to compare different signatures of species at each taxonomic level between treatments.

## 2 Results

### 2.1 Effects of exogenous organic acids and biological substrates on the alpha diversity of soil bacteria in mudflat

**Figure 1:**
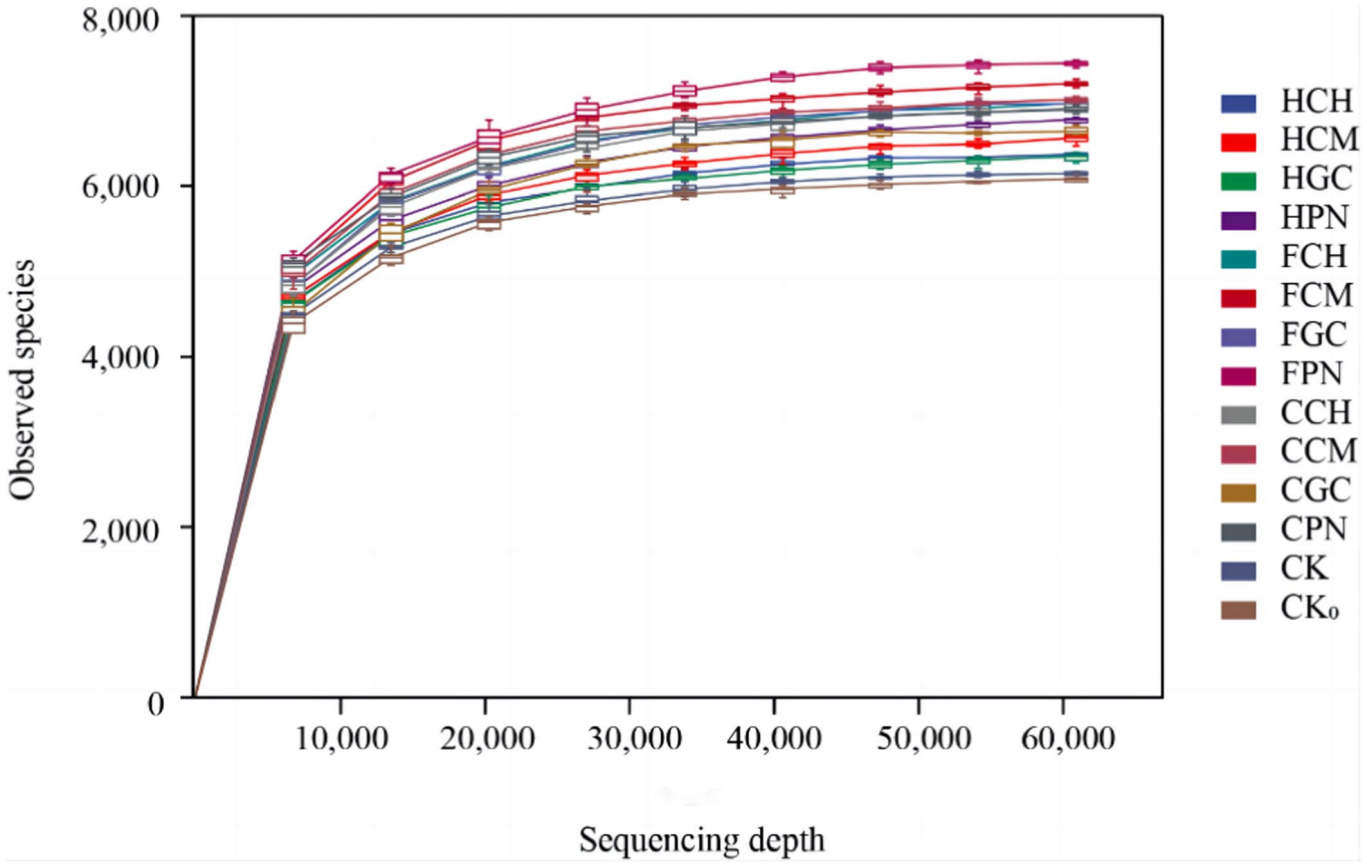
Rarefaction curves of soil bacterial sequences from different treatments.

The sparse curves of soil bacterial sequences under different treatments of exogenous organic acids and biological substrates are shown in Figure 1. The number of ASVs in each treatment first increased sharply with sequencing depth and then gradually flattened out. This finding indicated that the sequencing depth of the samples met the requirements of the analysis, and it is sufficient to cover most of the bacterial information in the samples, which can truly reflect the structural characteristics of bacterial communities in soil under different treatment conditions.

**Figure 2:**
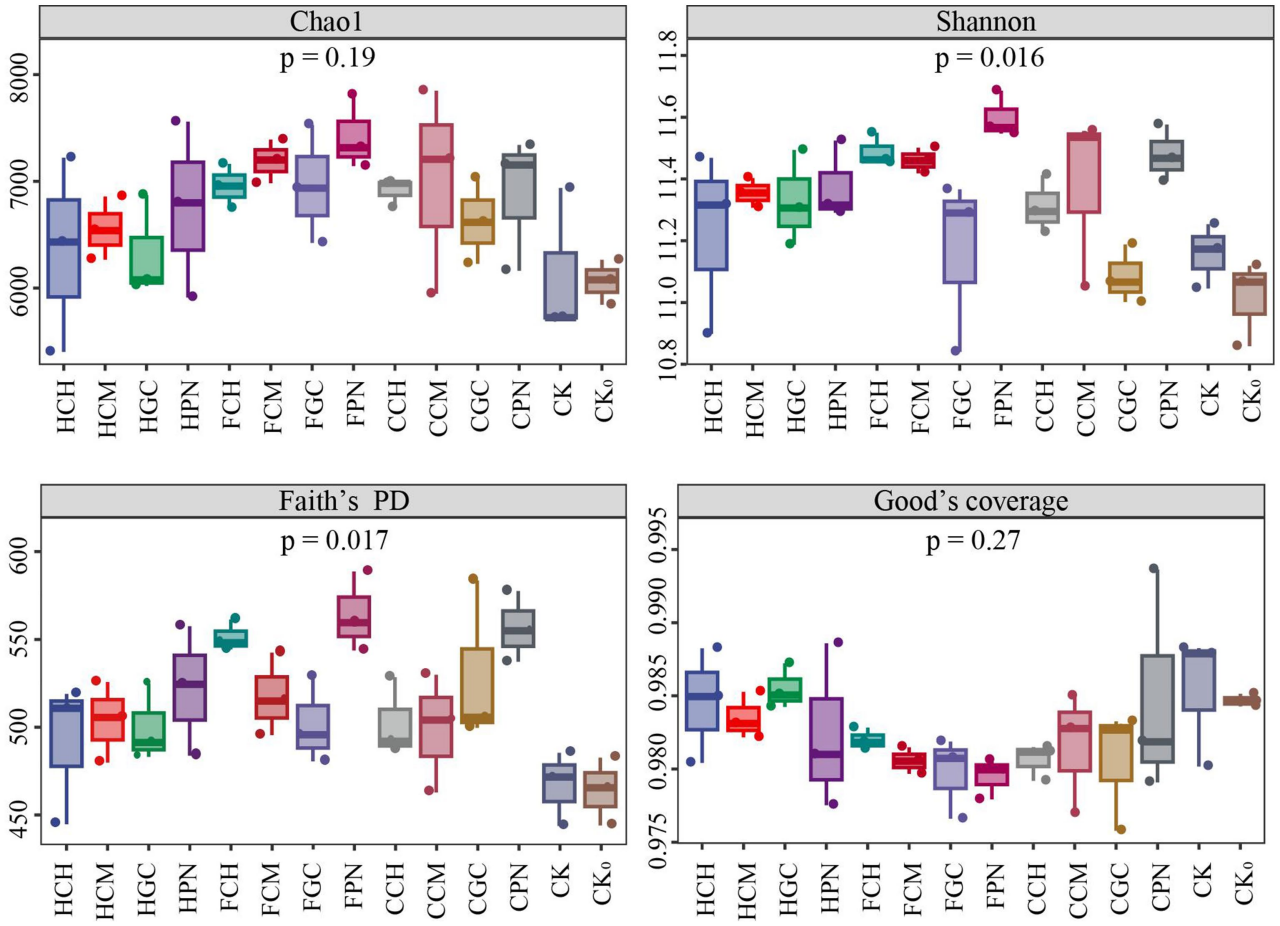
Effects of different treatments on the alpha diversity of bacterial communities in mudflat soil.

The effect of exogenous organic acids and biological substrates on the alpha diversity of soil bacterial community is shown in Figure 2. The average value of Chao1 index in descending order was FPN, FCM, CCM, FGC, FCH, CCH, CPN, HPN, CGC, HCM, HCH, HGC, CK, and CK_0_ treatments. The Chao1 index in the treatments of exogenous organic acids and biological substrates was higher than that in CK and CK_0_ treatments, indicating that the abundance of soil bacterial community under all treatments improved to varying degrees. The Shannon index in descending order was FPN, FCH, CPN, FCM, CCM, HPN, HCM, HGC, CCH, HCH, FGC, CK, CGC, and CK_0_ treatments. The bacterial diversity under treatments of exogenous organic acids and biological substrates, except CGC treatment, was higher than that of CK treatments. This finding indicated that the combination of citric acid and grass charcoal was not conducive for increasing the number of primitive bacterial species type in mudflat soils, whereas the other materials were favorable in increasing the community diversity. Faith’s PD index showed that all treatments with different exogenous organic acids and biological substrates contributed to the enhancement of the genetic diversity of bacterial community, with the FPN treatment having the highest effect. The Good’s coverage index of each treatment showed that the species coverage was in the range of 97.95–98.55%, which indicated that the experimental sampling was reasonable, and the sequencing volume was sufficient. Furthermore, the detection results can represent the real change rule of species diversity in bacterial communities.

### 2.2 Effects of exogenous organic acids and biological substrates on the beta diversity of soil bacteria in mudflat

**Figure 3:**
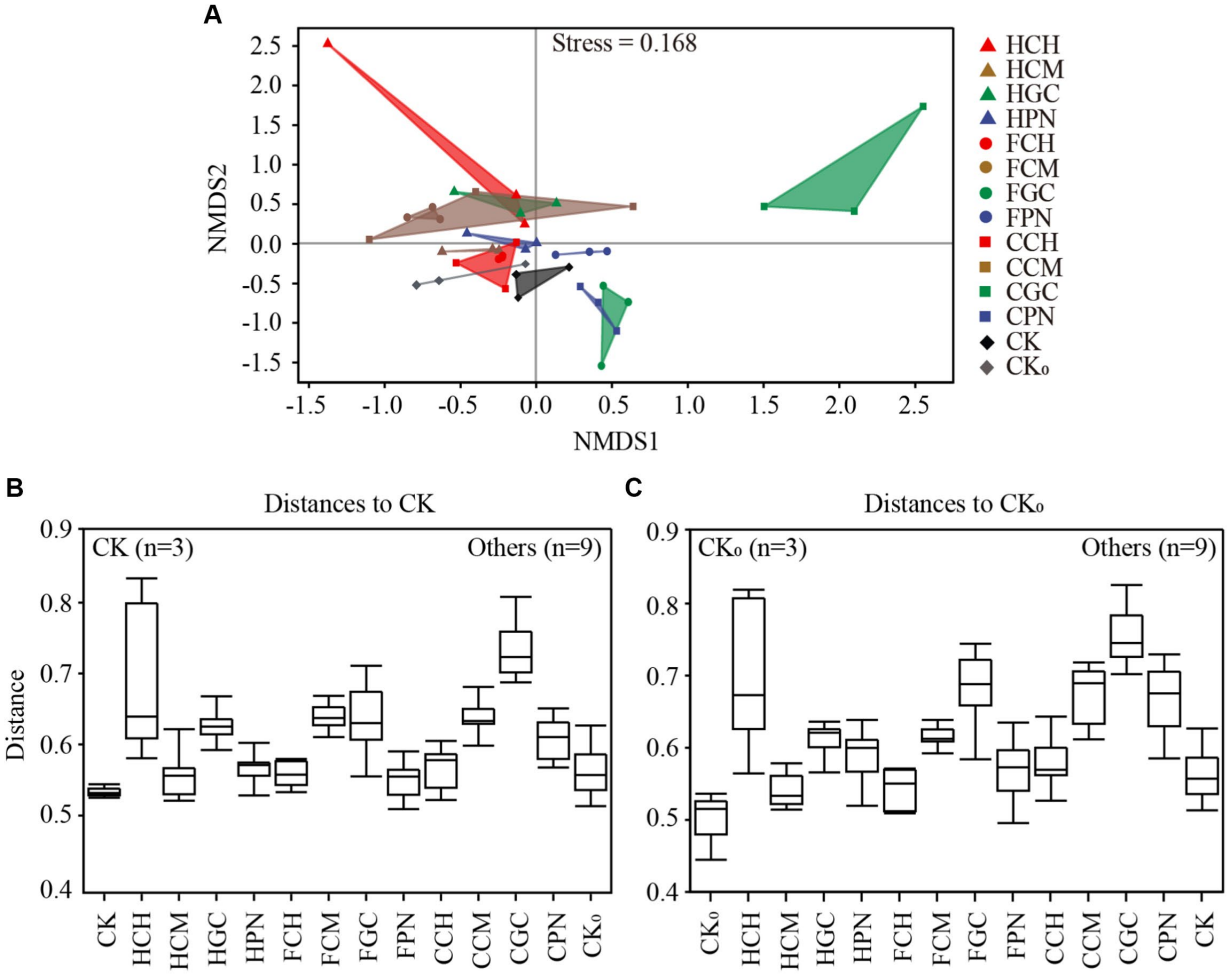
Analysis of group differences in bacterial communities of mudflat soil under different treatments. **(A)** shows the nonmetric multidimensional scaling analysis of soil bacterial communities in different treatments; **(B,C)** show the permutational multivariate analysis of variance of bacterial communities.

The NMDS analysis of soil bacterial community structure based on the abundance of ASVs is shown in Figure 3A. The stress value of the NMDS results was 0.168, indicating that the results of NMDS analysis can accurately reflect the true distribution of the data, and the closer the distance between the two points in the figure, the smaller the difference between the two bacterial communities. The distance among HCH, HGC, FCM, FGC, CCM, CGC, and CPN treatments was farther away than that in CK treatment, indicating that the difference in soil bacteria between them and CK treatment was more significant. Meanwhile, the distance among HCM, HPN, FCH, FPN, CK_0_, CCH, and CK treatments was relatively far away, indicating that the bacterial community structure was different under the above treatments compared with under CK treatment. However, the comprehensive effect on the changes in soil bacterial community structure was relatively small. In addition, the distance between CGC treatment and all other treatments was far, indicating that the structural composition of the soil bacterial community in CGC treatment was significantly different from those in other treatments. PERMANOVA, which is based on distance matrices, serves as a further computational test for NMDS analysis. It can determine specific differences between different treatments through pairwise comparisons. Figures 3B,C show the inter-group difference analysis of PERMANOVA based on CK and CK_0_ treatments compared with other treatments (*p* = 0.001). As shown in Figure 3B, the treatments that had the largest distances from CK treatments were CGC, HCH, FCM, CCM, FGC, HGC, and CPN treatments (*p* < 0.001). Figure 3C shows that the treatments with a large difference from CK_0_ treatment were CGC, CCM, FGC, HCH, CPN, HGC, and FCM treatments (*p* < 0.001). The calculation result was consistent with the distribution pattern presented by NDMS analysis, effectively verifying the significant difference in beta diversity among different treatments. These findings indicated that each exogenous organic acid composite and biological substrate had different influences on the structural composition of the bacterial community in mudflat soils.

### 2.3 Effect of exogenous organic acids and biological substrates community composition in mudflat soil

**Figure 4:**
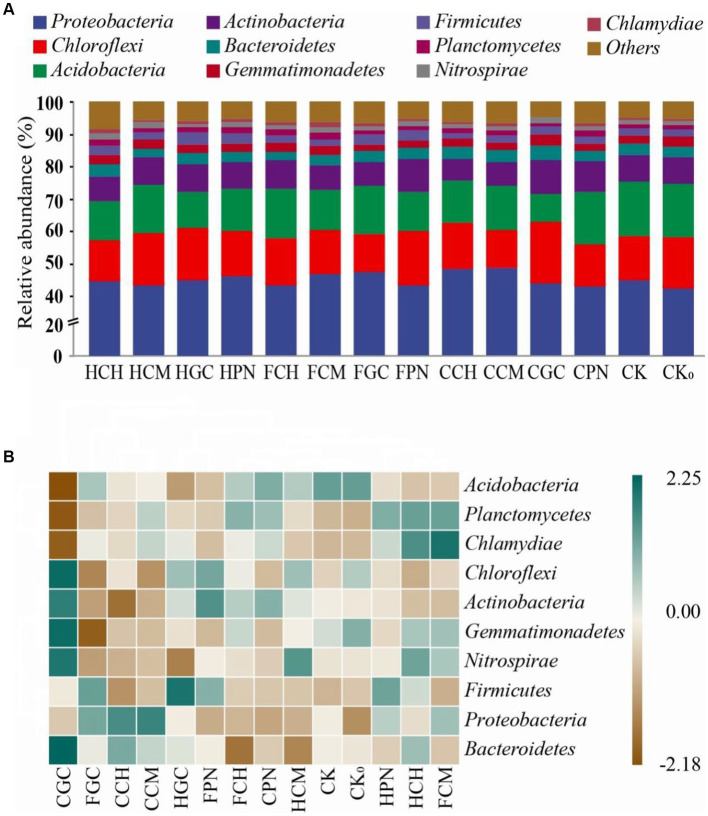
Community composition of top 10 bacterial phyla in terms of relative abundance in mudflat soil of different treatments. **(A)** shows the relative abundance of soil bacterial phyla in different treatments; **(B)** shows the clustering relationships among bacterial communities. The different color block sizes in **(A)** correspond to the relative abundance of each phylum; the blue and brown colors in **(B)** blue represent positive and negative correlations, respectively.

As shown in Figure 4, the top 10 bacterial phyla ranked in terms of relative abundance in soil bacterial communities at the phylum level were *Proteobacteria*, *Chloroflexi*, *Acidobacteria*, *Actinobacteria*, *Bacteroidetes*, *Gemmatimonadetes*, *Firmicutes*, *Planctomycotes*, *Nitrospirae*, and *Chlamydiae*. The soil bacterial community of different treatments was dominated by *Proteobacteria*, which accounted for 42.31–48.62% of the total number of bacteria, followed by *Chloroflexi*, *Acidobacteria*, and *Actinobacteria*, which accounted for 11.51–18.94%, 8.7–16.62%, and 6.4–10.44%, respectively. The bacteria whose relative abundance was in the range of 1–5% in different treatments were *Bacteroidetes*, *Gemmatimonadetes*, *Firmicutes*, *Planctomycotes*, and *Nitrospirae*, which accounted for 2.51–4.48%, 1.86–3.46%, 1.82–3.73%, 0.86–1.95%, and 1.08–1.96%, respectively, of the total bacterial population. Compared with that in CK treatment, the relative abundance of *Acidobacteria* in the treatment of exogenous organic acids and biological substrates decreased overall in the range of 3.37–47.62%, whereas the relative abundance of *Firmicutes*, *Planctomycotes*, and *Chlamydiae* significantly increased. FCM, CCM, HPN, FGC, and CCH treatments were conducive to the increase in the relative abundance of *Proteobacteria*; HCH, CCM, CPN, and FGC treatments caused a decrease in the relative abundance of *Chloroflexi*; and CPN, HGC, FCH, HCM, and FPN treatments facilitated an increase in *Actinobacteria*. The main bacteria in mudflat soil had different responses to the treatment of exogenous organic acids and biological substrates. Figure 4B shows the clustering relationship between treatments and bacterial communities at the phylum level. FPN and HGC, CCH and CCM, CPN and FCH, HCH and FCM, and CK and CK_0_ treatments were clustered to one another. This finding suggested that these treatments had similar effects on the soil bacteria, thereby showing a similar community composition. In addition, CGC treatment showed strong positive correlations with *Chloroflexi*, *Gemmatimonadetes*, and *Bacteroidetes* and strong negative correlations with *Acidobacteria*, *Planctomycotes*, and *Chlamydiae*. FGC treatment had a negative correlation with *Gemmatimonadetes*, CCH treatment had a negative correlation with the *phylum Actinobacteria*. FCM treatment had a positive correlation with *Chlamydiae*. The trends in the influence of various treatments with exogenous organic acids and biological substrates on the species composition and structural distributions of soil bacteria varied considerably.

**Figure 5:**
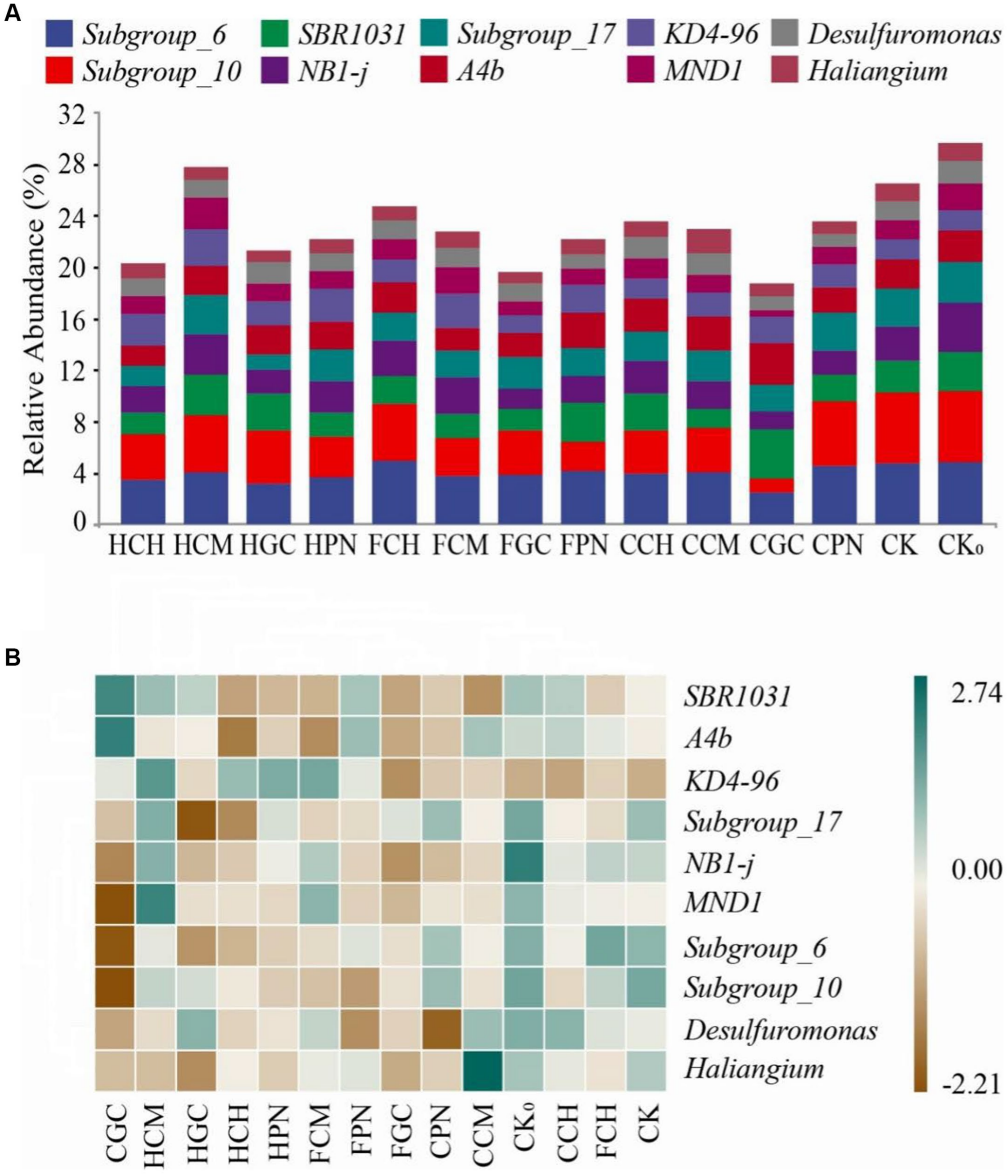
Community composition of top 10 bacterial genera in terms of relative abundance in mudflat soil of different treatments. **(A)** shows the relative abundance of soil bacterial genera in different treatments; **(B)** shows the clustering relationship between bacterial communities and treatments. The different color block sizes in **(A)** correspond to the relative abundance of each genus; the blue and brown colors in **(B)** blue represent positive and negative correlations, respectively.

Figure 5A shows that the ranking of the top 10 genera in soil bacterial community abundance at the genus level was as follows: *Subgroups_6*, *Subgroups_10*, *SBR1031*, *NB1-j*, *Subgroup_17*, *A4b*, *KD4-96*, *MND1*, *Desulfuromonas*, and *Hailiangium*. Among them, the relative abundance of *KD4-96* and *MND1* in HCM treatment was the highest, with increases of 79.11 and 66.89%, respectively, compared with that in CK treatment. The relative abundance of *SBR1031* and *A4b* in CGC treatment was the highest, with increases of 56.56 and 43.86%, respectively, compared with that in CK treatment. The genera *Subgroup_6* in FCH treatment and *Hailiangium* in CCM treatment exhibited the highest levels, with increases of 4.39 and 36.96%, respectively, compared with that in CK treatment. As shown in Figure 5B, HPN and FCM, FGC and CPN, and FCH and CK treatments were clustered to each other, indicating that the community structures showed a similarity. CGC treatment showed a strong positive correlation with *SBR1031* and *A4b* and a negative correlation with *MND1*, *Subgroup_6*, and *Subgroup_10*. CCM treatment showed a strong positive correlation with *Hailiangium*, CPN treatment showed a negative correlation with *Desulfuromonas*, and HGC treatment showed a negative correlation with *Subgroup_ 17*. These findings suggested that the dominant species at the genus level in mudflat soils under different treatments of exogenous organic acids and biological substrates differed considerably.

### 2.4 Analysis of soil bacterial ASVs and significant bacterial differences in treatments of exogenous organic acids and biological substrates

**Figure 6:**
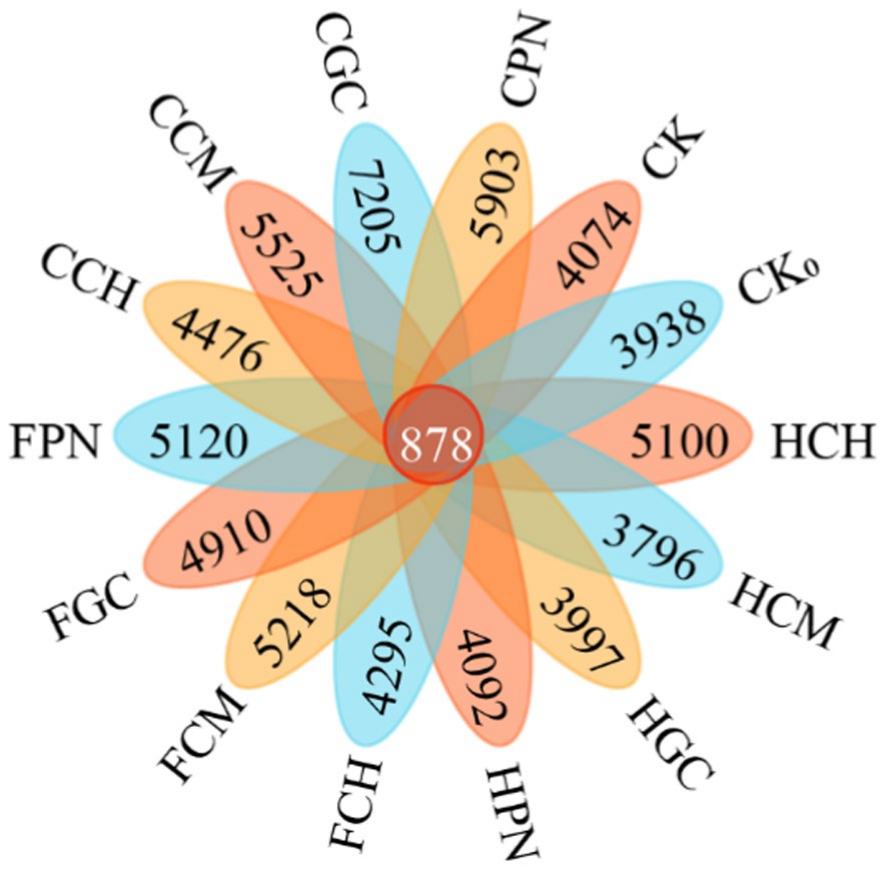
ASV numbers of bacterial community in mudflat soil under different treatments. HCH, humic acid–cottonseed hull composite; HCM, humic acid–cow manure composite; HGC, humic acid–grass charcoal composite; HPN, humic acid–pine needle composite; FCH, fulvic acid–cottonseed hull composite; FCM, fulvic acid–cow manure composite; FGC, fulvic acid–grass charcoal composite; FPN, fulvic acid–pine needle composite; CCH, citric acid–cottonseed hull composite; CCM, citric acid–cow manure composite; CGC, citric acid–grass charcoal composite; CPN, citric acid–pine needle composite; CK without the addition of organic acids and biological substrates; CK_0_, bare area. The same for the following.

Figure 6 shows that except for HCM and HGC treatments, all treatments of exogenous organic acids and biological substrates improved the soil bacterial community compared with CK treatment, and the number of ASVs of the 14 treatments in descending order was as follows: 7205, 5,903, 5,525, 5,218, 5,120, 5,100, 4,910, 4,476, 4,295, 4,092, 4,074, 3,997, 3,938, and 3,796 in CGC, CPN, CCM, FCM, FPN, HCH, FGC, CCH, FCH, HPN, CK, HGC, CK_0_, and HCM treatments, respectively. The number of shared ASVs was 878. The enhancement of soil bacterial ASV by different treatments of exogenous organic acids and biological substrates varied significantly, with CGC, CPN, CCM, FCM, FPN, HCH, FGC, CCH, FCH, and HPN treatments showing increases of 76.85, 44.89, 35.62, 28.08, 25.68, 25.18, 20.52, 9.87, 5.42, and 0.44%, respectively, compared with CK treatment. On the contrary, HGC, CK_0_, and HCM treatments had decreases of 1.89, 3.34, and 6.82%, respectively.

**Figure 7:**
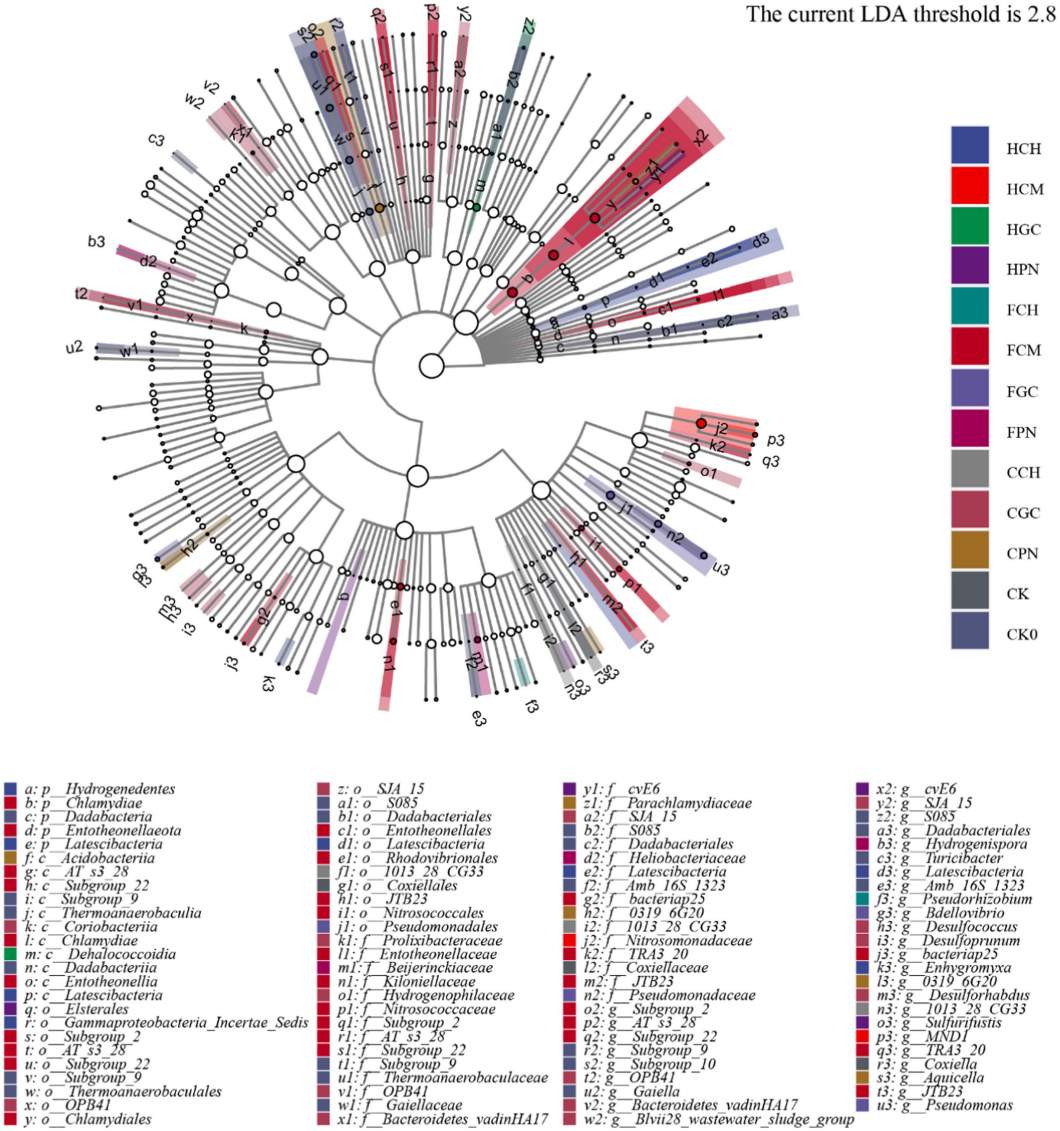
LEFSe analysis of significantly different bacteria in mudflat soil among different treatments. The branches from the inner circle to the outer circle show the taxonomic rank relationships of soil bacterial communities at phylum, class, order, family, and genus levels. The node size corresponds to the mean relative abundance of the taxon. The hollow nodes represent taxa with insignificant between-treatment differences. The colored nodes indicate that these taxa have significant between-treatment differences and that the abundance is high in the treatment represented by the color.

The LEFSe of significantly different bacteria in the bacterial community of mudflat soil treated with exogenous organic acids and biological substrates was analyzed at different taxonomic levels, and the results are shown in Figure 7. A total of 33 significantly different bacteria were found among the 14 treatments, The LEFSe of significantly different bacteria in the bacterial community of mudflat soil treated with exogenous organic acids and biological substrates was analyzed at different taxonomic levels, and the results are shown in Figure 7. A total of 33 significantly different bacteria were found among the 14 treatments. At the genus level, the FCM treatment contained the highest number of characterized genera with significant differences, seven (*OPB41*, *Bacteroidetes_vadinHA17*, *Blvii28_wastewater_sludge_group*, *SJA_15*, *Desulforhabdus*, *Desulfococcus*, *Desulfoprunum*); FCM (*bacteriap25*, *TRA3_20*, *JTB23*, *subgroup_2*, *AT_s3_28*, *Subgroup_22*) and CK_0_ (*Subgroup_9*, *S085*, *Dadabacteriales*, *Turicibacter*, *Amb _16S_1323*, *Gaiella*) contain six characterized genera; HCH (*Latescibacteria* and *Enhygromyxa*), HPN (*cvE6* and *Sulfurifustis*), FGC (*Pseudomonas* and *Bdellovibrio*), CPN (*0319_6G20*, *Aquicella*), CK (*Coxiella* and *Subgroup_10*) contained two characteristic genera. HCM, HGC, FCH, FPN, CCH contained one characteristic genus, *MND1*, *S085*, *Pseudorhizobium*, *Hydrogenispora*, *1013_28_CG33*; CCM No characteristic genera containing significant differences were detected.

## 3 Discussion

### 3.1 Effects of exogenous organic acids and biological substrates on bacterial community diversity in mudflat soil

Soil bacterial community is sensitive to changes in physicochemical factors, and it has a certain preference for organic materials from different sources. Therefore, after long-term improvement of soil by using organic materials, some bacteria could gradually enrich and lead to a change in the composition and diversity of the original bacterial community (Canellas et al., 2015). The present study showed that the alpha diversity of bacterial community richness and genetic diversity under the treatment of different exogenous organic acids was higher than those under CK and CK_0_ treatments, but the difference was not significant. The results of beta diversity showed that the bacterial community structure under different treatments changed significantly, which was similar to the change rule of Shi et al. (2018), who used organic acids to improve microbial community structure in saline soil. Low molecular weight organic acids in the soil participate in soil formation, promote mineral dissolution, and change soil physicochemical properties, thereby alleviating the toxic effects of elements such as metals on plants or soil microorganisms (Lima et al., 2009; Gao et al., 2015). It can also mediate interactions between plants and soil microorganisms, increase soil enzyme activity and microbial activity, promote the formation of aggregates, and accelerate soil nutrient cycling to improve the soil ecosystem. It was found that exogenously applied organic acids can adjust the acid – base and redox conditions of soil tillage using acidic functional groups, adsorb soil saline ions through chelation, and enhance the solubility and mobility of phosphorus, thus promoting the enhancement of effective nutrients in saline soils (Chatterjee et al., 2015). Consequently, it may be due to the addition of exogenous substances to improve the soil microbial survival of the adverse environmental conditions, such as soil structure, pH, aeration and permeability, and topsoil nutrient composition changes (Yang et al., 2023), the improvement of the adverse environmental conditions makes most of the bacterial community’s more easy to grow and reproduce, which adjusts the species and number of various bacterial groups in the soil, resulting in the bacterial community development direction differences, changing the diversity of the community. However, short-term application cannot easily have a significant effect on bacterial community diversity due to the relatively stable structure of organic materials (Wu et al., 2013).

The bacterial community in this study had a positive response to the addition of citric acid complex, with an increase in bacterial relative abundance and community diversity. Similarly, Bao et al. (2022) used exogenous citric acid to improve the removal effect of polycyclic aromatic hydrocarbons (PAHs) in contaminated soil. Meanwhile, the fulvic acid component of the composite was more effective in increasing the diversity of the bacterial community of cultivated soils than citric acid, similar to the study results of Li et al. (2021) and Sun et al. (2023) on fulvic acid organic fertilizers. The alpha diversity of the bacterial community was the highest in FPN treatment, followed by CCM. The main reason for the difference in the response of soil bacterial community in mudflat to fulvic acid–pine needles and citric acid–cow manure is that fulvic acid is highly soluble in alkaline environments and has a microporous structure accessible to microorganisms, thus attracting a large number of bacteria attached to the surface of the soil colloid to come close to it (Zhang et al., 2020). When combined with pine needles with high nutrient balance and total amount, fulvic acid can adapt to the feeding preferences of different kinds of microorganisms, with a wide range of energy supply, which is conducive to the formation of microbial diversity (Wang et al., 2011). Meanwhile, the high C/N ratio of citric acid can provide abundant and available carbon sources for bacteria, thus promoting the life activities and nutrient turnover of soil bacteria (Chen et al., 2008). Cow manure contains more cellulose and lignin, which can be slowly decomposed by specific bacterial communities in aerobic or anaerobic soil environments, thereby supporting the increase in its population (Meng et al., 2019). In summary, the addition of exogenous organic acids and biological substrates provides a relatively independent microenvironment and sufficient nutrients for the life activities of different energy-type bacteria, thereby playing a regulatory role in the species composition and functional characteristics of bacterial communities. Furthermore, it has a significant effect on the overall diversity of bacterial communities.

### 3.2 Effect of exogenous organic acids and biological substrates community composition in mudflat soil

In this study, the main bacteria with relative abundance greater than 5% in the bacterial community of mudflat soil were *Proteobacteria*, *Chloroflexi*, *Acidobacteria*, and *Actinobacteria*, with *Proteobacteria* having absolute dominance in all treatments, consistent with the results of most studies on bacterial diversity in saline alkaline soil environments (Canfora et al., 2014; Abdallah et al., 2018; Ayantha et al., 2018). The highest relative abundance of *Proteobacteria* in CCM treatment may be due to the fact that the increased level of soil humus caused by the application of fermented cow manure positively affected the increase in the number of *Proteobacteria*, a group of eutrophic bacteria, and resulted in a relative decrease in the proportion of nutrient-poor bacteria in the soil (Liu et al., 2021). Moreover, a large number of *Proteobacteria* contributed to soil carbon storage through the synthesis of microbial mucilage and polysaccharides that contribute to the stabilization of aggregates in mudflat soils (Pascual et al., 2018). The high relative abundance of *Chloroflexi* in FPN treatment was attributed to the higher nitrogen content of fulvic acid and pine needles than other materials; such a high content attracts the migration and colonization of *Chloroflexi* to the tillage layer, facilitates nitrification of the soil in the root zone, and replenishes the nitrogen deficit (Zhao et al., 2014). The highest relative abundance of *Actinobacteria* in CGC treatment was attributed to its ability to produce various extracellular hydrolases, which degrade and convert exogenous organic matter in the soil into soluble phosphorus, nitrogen, potassium, and other components and play an important role in the mineralization of organic matter. The addition of citric acid and grass charcoal was beneficial to the propagation and metabolic activities of *Actinobacteria*, similar to the effect of organic materials on the microbial community of alkalized soil in the study of Liang et al. (2023). In the present study, the treatment of organic acids and biological substrates resulted in a decrease in the relative abundance of native *Acidobacteria* in saline alkali soil. Given that *Acidobacteria* is an oligotrophic bacterium, nutrients have an important influence on its lifestyle, and excess nutrients reduce its activity and thus lead to a decrease in abundance (Ward et al., 2009).

The dominant genera of soil bacteria in this study belong to *Acidobacteria*, *Chloroflexi*, and *Proteobacteria*, respectively. These genera are more tolerant in alkaline soils, with certain degradation functions for complex compounds. The increase in the relative abundance of these genera is favorable to the nutrient cycling and supply of plant rhizosphere soil, and they belong to the bacterial communities that widely exist in pristine saline and alkaline soils and have a high application value for saline and alkaline soil remediation (Liu et al., 2016). The results of the present study showed that the signature differential bacterial species and quantities of bacterial communities varied among different treatments of organic acids and biological substrates, because the physicochemical properties of the exogenous materials are an important driver of the variability in the structure of the soil bacterial community. These properties not only influence the relative abundance of dominant species of the bacterial community but also utilize the trophic relationships of bacteria to promote the emergence of bacterial endemics, in agreement with the findings of Jung and Choi (2020). In summary, the addition of exogenous organic acids and biological substrates can cause changes in the original bacterial community composition of mudflat soil. However, this change can take a long time to maintain to form a stable bacterial community structure. The effect of exogenous organic acids and biological substrates on the specific function of soil bacterial community needs to be further explored, and the available bacterial resources of mudflat soil need to be further revealed.

## 4 Conclusion

The results of the study on the effect of exogenous organic acid composite biological substrates additions on the structural characteristics of bacterial communities in beach soils showed that different treatments of exogenous organic acids and biological substrates had no significant effect (*p* ≥ 0.05) on bacterial alpha diversity, but FPN treatment showed a slight increase in the alpha index values compared with CK treatment. Significant differences (*p* < 0.05) were found in the beta diversity of bacterial communities among all treatments, with the greatest difference found in CGC treatment. CCM, FPN, and CGC treatments increased the relative abundances of *Proteobacteria*, *Chloroflexi*, and *Actinobacteria*, respectively, thus playing important roles in nutrient cycling and supply in mudflat soils.

## Data availability statement

The datasets presented in this study can be found at https://www.ncbi.nlm.nih.gov under accession number SRP498511.

## Author contributions

XYL: Data curation, Formal analysis, Writing – original draft, Writing – review & editing. LZ: Writing – original draft. RY: Conceptualization, Resources, Writing – original draft. HW: Supervision, Validation, Writing – review & editing. XBL: Methodology, Supervision, Writing – review & editing. WX: Supervision, Validation, Writing – review & editing. HY: Supervision, Writing – review & editing. YS: Methodology, Supervision, Writing – review & editing. JL: Writing – review & editing. ZS: Funding acquisition, Project administration, Resources, Visualization, Writing – review & editing, Writing – original draft.
