# Supplementary figures and images for: Modifying soil bacterial communities in saline mudflats with organic acids and substrates
Source: Front Microbiol. 2024 Apr 19;15:1392441. doi: 10.3389/fmicb.2024.1392441 (PMC11066327; doi:10.3389/fmicb.2024.1392441)

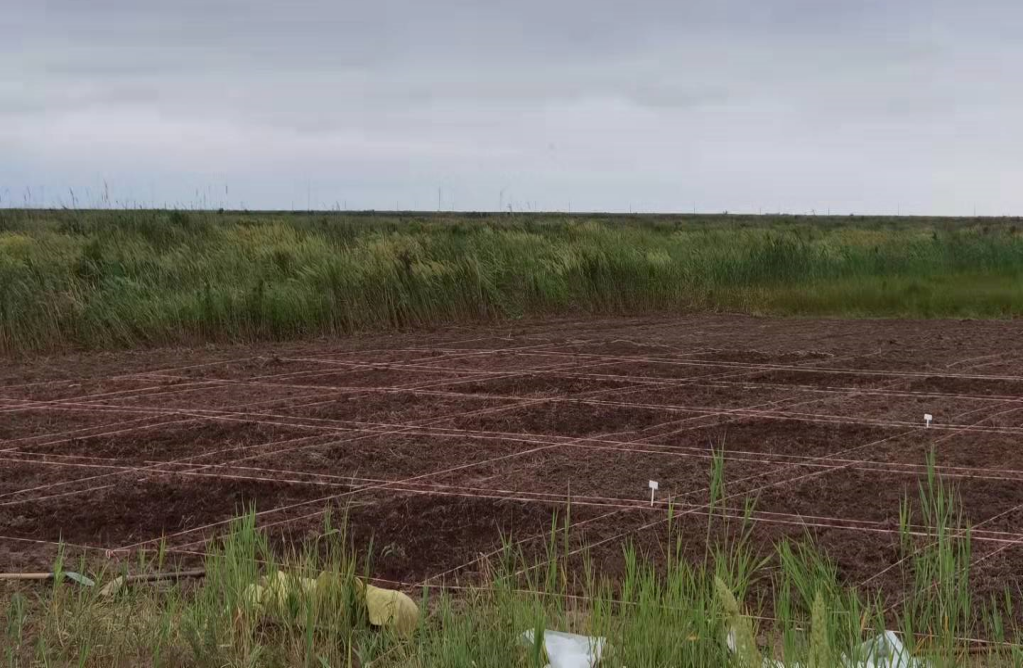

Supplement: Supplementary file 1 [file Image_1.PNG]

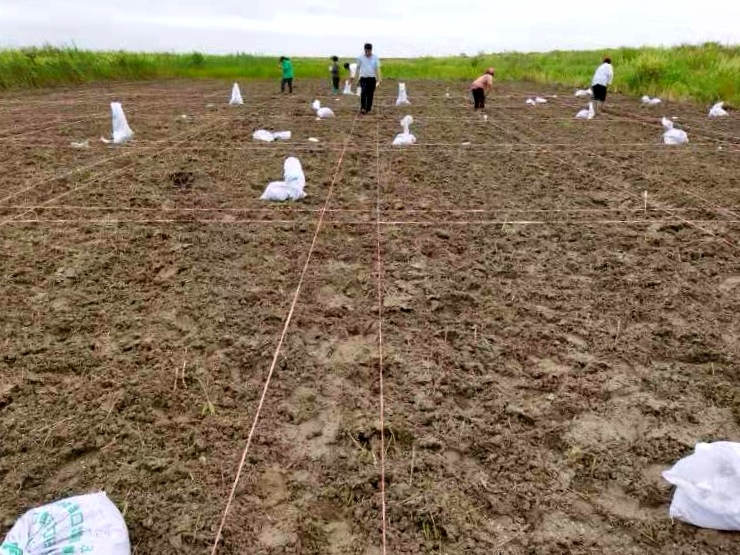

Supplement: Supplementary file 2 [file Image_2.PNG]

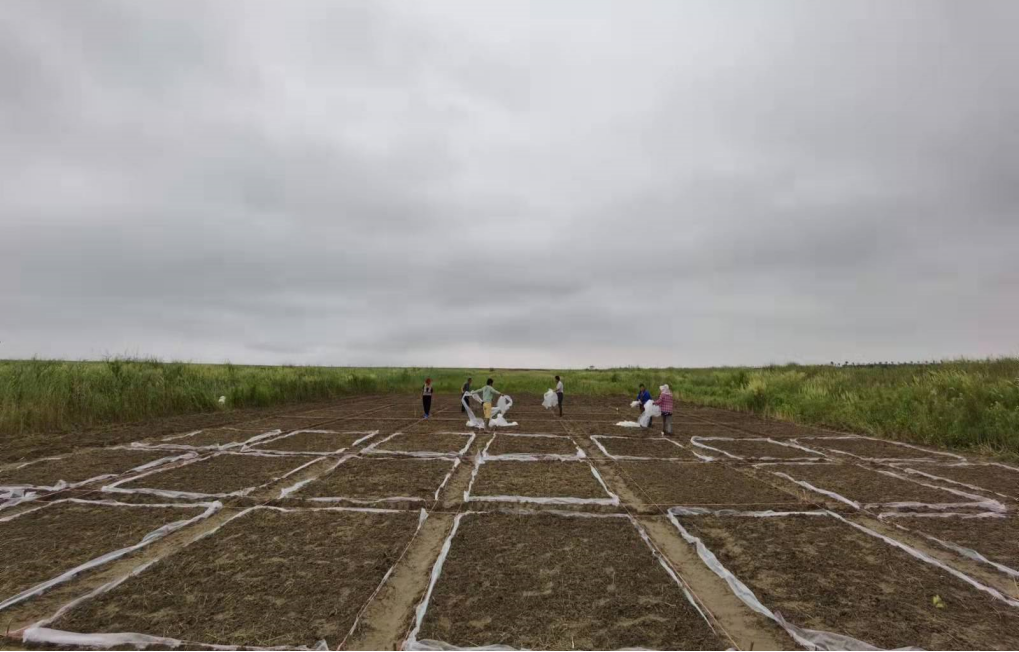

Supplement: Supplementary file 3 [file Image_3.PNG]
